# Comparable cognitive impairment was detected in MACS and CS and alleviated after remission of hypercortisolism in MACS

**DOI:** 10.3389/fendo.2024.1373101

**Published:** 2024-07-31

**Authors:** Mengsi Liu, Wenji Zhao, Wei Zhang, Zhaoyang Tian, Zhou Zhang, Yuan Lou, Ziwei Zhang, Fan Yang, Dalong Zhu, Ping Li

**Affiliations:** ^1^ Department of Endocrinology, Endocrine and Metabolic Disease Medical Center, Nanjing Drum Tower Hospital, Affiliated Hospital of Medical School, Nanjing University, Nanjing, China; ^2^ Department of Endocrinology, Endocrine and Metabolic Disease Medical Center, Nanjing Drum Tower Hospital, Chinese Academy of Medical Sciences & Peking Union Medical College, Graduate School of Peking Union Medical College, Nanjing, China; ^3^ Nanjing Drum Tower Hospital, Branch of National Clinical Research Centre for Metabolic Diseases, Nanjing, China

**Keywords:** adrenal incidentalomas, mild autonomous cortisol secretion, Cushing’s syndrome, cognitive impairment, adrenalectomy

## Abstract

**Context:**

Few studies have directly compared the cognitive characteristics of patients with mild autonomous cortisol secretion (MACS) and Cushing’s syndrome (CS). The effect of surgical or conservative treatment on cognitive function in patients with MACS is still unclear.

**Objective:**

To compare the differences in cognitive function between patients with MACS and CS and evaluate the effect of surgery or conservative treatment on cognitive function.

**Methods:**

We prospectively recruited 59 patients with nonfunctional adrenal adenoma (NFA), 36 patients with MACS, and 20 patients with adrenal CS who completed the global cognition and cognitive subdomains assessments. Seventeen MACS patients were re-evaluated for cognitive function after a 12-month follow-up period; of these, eleven underwent laparoscopic adrenalectomy and six received conservative treatment.

**Results:**

Patients with MACS and CS performed worse in the global cognition and multiple cognitive domains than those with NFA (all P<0.05). No statistical difference was found in cognitive functions between patients with MACS and CS. Logistic regression analysis showed that patients with MACS (odds ratio [OR]=3.738, 95% confidence intervals [CI]: 1.329–10.515, P=0.012) and CS (OR=6.026, 95% CI: 1.411–25.730, P=0.015) were associated with an increased risk of immediate memory impairment. Visuospatial/constructional, immediate and delayed memory scores of MACS patients were significantly improved at 12 months compared with pre-operation in the surgical treatment group (all P<0.05), whereas there was no improvement in the conservative treatment group.

**Conclusion:**

Patients with MACS have comparable cognitive impairment as patients with CS. Cognitive function was partially improved in patients with MACS after adrenalectomy. The current data support the inclusion of cognitive function assessment in the clinical management of patients with MACS.

## 1 Introduction

Adrenal incidentalomas (AI) are commonly defined as clinically unapparent adrenal masses found incidentally during imaging performed for indications other than the suspected adrenal disease (1). With the increased use of imaging, the incidence of AI has increased by 10-fold over the past two decades (2). Approximately 30% patients with AI are found to have mild autonomous cortisol secretion (MACS), a subtle form of cortisol excess (1). The difference between overt Cushing’s syndrome (CS) and MACS is the severity of biochemical and physical manifestations. Although to a lesser extent, the presence of MACS has also been associated with increased risk of diabetes, hypertension, obesity, osteoporosis, as well as increased cardiovascular morbidity and mortality (3–5).

Cognitive impairment is a common finding in patients with CS and severely impairs patients’ quality of life (6). Memory was the most commonly affected domain (83% cases) (7). Other cognitive impairments in the domains of attention, visuospatial functioning, and language functioning have also been documented (7, 8). However, previous studies have found that cognitive function impairment in patients with CS did not improve in the short-term (9). Even in the long-term follow-up, there is only partial normalization of cognitive function, suggesting that chronic hypercortisolemia causes irreversible adverse effects on the central nervous system (9, 10). When the abundance of glucocorticoid receptors in the brain is considered, it is tempting to speculate that brain function abnormalities found in patients with CS also apply to patients with MACS. In our recently published study (11), we reported for the first time that patients with MACS showed evidence of impaired cognitive function. Our study showed that patients with MACS performed worse in working memory and visuospatial/constructional domains than those with non-functioning adrenal adenomas (NFA) (11). Up till now, few studies have carried out direct comparisons of cognitive function between patients with MACS and CS. Furthermore, the effect of surgical or conservative treatment on cognitive function in patients with MACS is still unclear.

Therefore, we performed a prospective study to assess whether the anticipated differences in cognitive functions between patients with MACS and those with CS are significant. Additionally, we carried out a longitudinal study to evaluate the cognitive alterations in MACS patients one year after surgical treatment or conservative treatment to explore whether cognitive impairment can be reversed or ameliorated after the remission of hypercortisolism in MACS.

## 2 Patients and methods

### 2.1 Study population

We conducted a prospective study at the Department of Endocrinology of Nanjing Drum Tower Hospital from October 2021 to September 2023, on a consecutive series of inpatients with AI and suspected overt CS. All the study procedures were approved by our institutional research and ethics review board and registered on Clinical-Trials.gov (NCT05357456). All participants signed an informed consent form, and the study was conducted in accordance with the tenets of the Helsinki Declaration.

**Figure 1:**
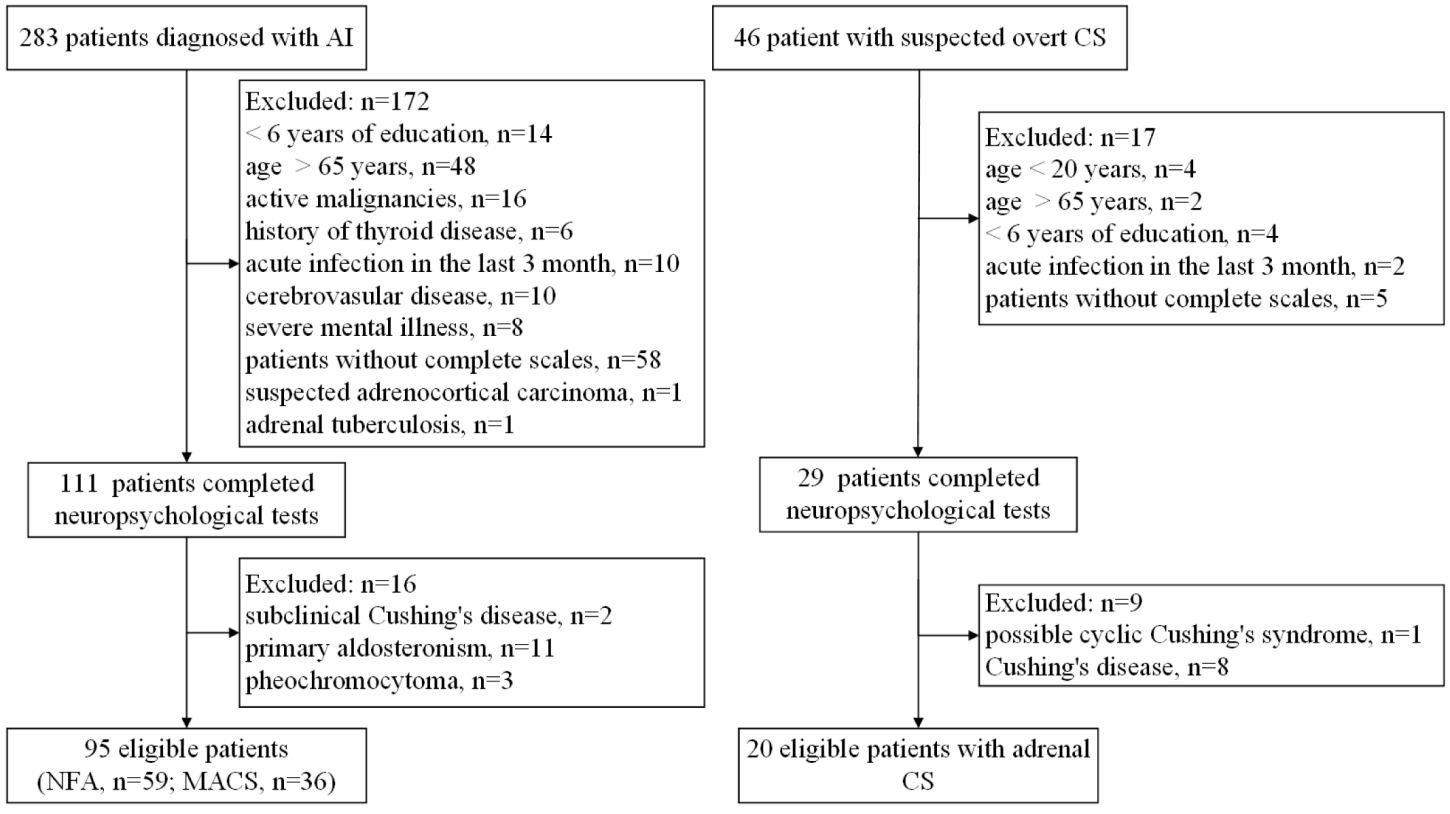
Flow diagram of patients recruited for this study. AI, adrenal incidentalomas; NFA, nonfunctional adrenal adenoma; MACS, mild autonomous cortisol secretion; CS, Cushing’s syndrome.

Eligible participants with AI and suspected overt CS were aged between 20 and 65 years and had attained at least 6 years of education. Patients were excluded if they (i) had factors that affected cognitive function (history of malignancy, hypothyroidism, hyperthyroidism, and acute infection in the last 3 months, history or presence of severe neurological and/or psychiatric illness, drug, and/or alcohol abuse); (ii) were unable to undergo complete neuropsychological testing; (iii) were diagnosed with suspected adrenocortical carcinoma, adrenal tuberculosis, subclinical Cushing’s disease, primary aldosteronism, pheochromocytoma, Cushing’s disease, iatrogenic Cushing’s syndrome, or cyclic Cushing’s syndrome; (iv) had other confounding factors (e.g., severe impairment of heart, liver, kidney, and other organs, pregnancy, or lactation). A total of 283 patients with AI and 46 patients with suspected overt CS were initially evaluated. Of these, neuropsychological tests were conducted on 111 patients with AI and 29 patients with overt CS. After further exclusion criteria, 59 patients with NFA, 36 patients with MACS and 20 patients with adrenal CS were eventually enrolled. The number of patients included and excluded, along with the reasons for exclusion, are presented in **Figure 1**.

### 2.2 Clinical evaluation

We implemented standardized radiological assessments and systematic hormone assessments of patients with AI and suspected overt CS as recommended by the internationally agreed guidelines (1, 12). Demographic information, detailed patient history, and medication data were collected on standardized questionnaires at enrollment. Body weight, height, and blood pressure were measured according to a standard protocol. Diagnosis of overweight or obesity, impaired glucose regulation (IGR) or type 2 diabetes mellitus (T2DM), hypertension, dyslipidemia, osteopenia, or osteoporosis was based on current standards (13–17).

### 2.3 Hormonal evaluation

All patients with AI were evaluated for the following by measuring: (i) plasma adrenocorticotropic hormone (ACTH) levels and morning serum cortisol levels at 0800 h and 2400 h; (ii) 24-h urinary free cortisol (UFC); (iii) serum dehydroepiandrosterone sulfate; (iv) serum cortisol levels after 1 mg dexamethasone suppression test (DST); and (v) plasma-fractionated metanephrines, plasma renin activity, and serum aldosterone concentration. Apart from the aforementioned evaluations, patients with suspected overt CS were further evaluated for serum cortisol levels after low-dose DST (LDDST) and localization tests, including high-dose DST, CT of adrenal adenoma, MRI of pituitary gland, and inferior petrosal sinus sampling if necessary. For the 1 mg DST, the patient took 1.0 mg of dexamethasone at 12:00 am (midnight), and the cortisol levels were measured at 8:00 am the following morning. In the LDDST, the patient took 0.5 mg of dexamethasone every 6 hours for 48 hours, and the serum cortisol was measured the following morning at 8:00 am.

### 2.4 Clinical definitions

NFA was defined as a condition with a normally suppressed cortisol level of ≤50 nmol/L after 1 mg DST without primary aldosteronism and pheochromocytoma. We diagnosed MACS by the absence of symptoms and/or signs of overt hypercortisolism and by the presence of serum cortisol >50 nmol/L after 1 mg DST (1). The diagnosis of adrenal CS were based on the presence of overt clinical manifestations (i.e., abdominal purple striae, proximal muscle weakness, fragile skin with bruising, and moon facies), abnormal biochemical tests (elevated midnight serum cortisol and 24-h UFC levels, suppressed morning plasma ACTH, and serum cortisol ≥50 nmol/L after LDDST), and localization tests confirming adrenal adenoma or nodules (12).

### 2.5 Cognitive function assessments

The procedures implemented to assess cognitive function have been prescribed in our previously published work (11). The evaluation began with an interview obtaining information regarding the patient’s current memory-related complaints and relevant background information. Global cognition and multiple cognitive subdomains were assessed using the Chinese version of the Mini-Mental State Examination (MMSE), the Beijing version of the Montreal Cognitive Assessment (MoCA), and the Repeatable Battery for the Assessment of Neuropsychological Status (RBANS) (18, 19). The RBANS which is an effective screening tool for a wide range of neurocognitive abilities (20), contains 12 subtests that provide five index scores, namely immediate memory, visuospatial/constructional, language, attention, and delayed memory. Combining these index scores provides an overall performance score, with lower scores indicating worse cognitive function (19). Working memory, processing speed, and executive function cognitive domains were also assessed. Mild cognitive impairment (MCI) was identified by using education-specific cut-off points of the total MoCA scores (<26) (21). A score of <85 points in a specific subdomain in RBANS was considered a probable cognitive impairment in that domain (22). All tests were conducted by a well-trained physician in a separate room. The whole cognitive assessment testing took approximately 30–40 min to complete.

### 2.6 Treatment and follow-up

Surgery or conservative management was suggested for MACS patients according to age, degree of cortisol excess, general condition, comorbidities related to autonomous cortisol secretion, and patient preference (1). Briefly, 16 patients with MACS underwent laparoscopic unilateral adrenalectomy and 20 patients with MACS selected conservative management. A stress dose of hydrocortisone was intravenously administered during the postoperative period according to the institutional routine protocol. At discharge, patients usually received 20–30 mg hydrocortisone per day, and this dose was tapered by 2.5–5 mg/day every 2–4 weeks according to the patients’ complaints (i.e., poor appetite, nausea, vomiting, and fatigue), and the results of morning plasma ACTH and serum cortisol levels. Seventeen of 36 patients were re-evaluated for cognitive function after a 12-month follow-up period. At the time of the cognitive function assessment, all surgical patients with MACS had discontinued steroid replacement therapy for more than 3 months.

### 2.7 Laboratory evaluation

Serum cortisol and 24-h UFC levels were measured by direct chemiluminescent immunoassay using Atellica (Siemens Company, USA). The reference range for the serum cortisol levels was 145.4–619.4 nmol/L in the morning (0700–0900 h) and 94.9–462.4 nmol/L in the afternoon (1500–1700 h). Plasma ACTH concentrations were measured by chemiluminescence immunoassay (Siemens Healthcare Diagnostics, Shanghai, China) using Immulite 2000xpi (Siemens, UK) with the reference range of 0–10 pmol/L for morning plasma ACTH concentration. The inter- and intra-assay CVs were <6.25% and <8.33%, respectively.

### 2.8 Statistical analysis

All data were tested for normality with the Shapiro–Wilk test. Variables were expressed as the mean ± standard deviation for normally distributed data, median (25th percentile, 75th percentile) for non-normally distributed data, and as absolute numbers or percentages (%) for categorical data. Continuous variables among the three groups were compared using one-way analysis of variance (ANOVA) with *post-hoc* Bonferroni correction or Dunnett T3 tests, while categorical variables were compared using the χ^2^ or Fisher’s exact test. For non-normally distributed data, the Mann–Whitney U test or Kruskal–Wallis test was used as appropriate. The cognitive function among patients with NFA, MACS and CS, as well as among patients with MACS who opted for conservative and surgical treatment at baseline, was compared using analysis of covariance (ANCOVA) adjusted for age, sex and years of education, and after applying Bonferroni correction. Logistic regression models were constructed to estimate the odds ratio (OR) and 95% confidence interval. Paired T-test was used to compare the differences in cortisol-related hormone levels and neurocognitive scale scores between the conservative and the surgical treatment group at baseline and 12-month follow-up. ANCOVA was used to compare the differences in neurocognitive scale scores among MACS patients after a 12-month follow-up between the conservative and the surgical treatment groups, with adjustment for baseline values and age. All statistical analyses were performed using SPSS version 26.0 and GraphPad Prism version 9.0. A two-sided *P*<0.05 was considered significant.

## 3 Results

### 3.1 Clinical and endocrine characteristics of the study population

The study population comprised 115 patients (NFA: n=59; MACS: n=36; CS: n=20). **Table 1** summarizes the clinical and endocrine characteristics of the three study groups. The age, years of education and body mass index of the three groups were comparable (all *P*>0.05). The proportion of women and tumor diameter in the CS group were both higher than those in the NFA group, with no significant statistical difference observed between the CS group and the MACS group. With respect to comorbidities, the CS group exhibited a higher proportion of overweight/obesity, IGT/T2DM, hypertension, dyslipidemia, and osteopenia/osteoporosis than the NFA and MACS groups, but these differences were not statistically significant. The above metabolic comorbidities were similar in the MACS and NFA groups. As for hormone levels, morning plasma ACTH and DHEAS levels in the MACS group were significantly lower than those in the NFA group, while midnight serum cortisol and cortisol levels after 1 mg DST were higher than those in NFA group (all *P*<0.05). No significant differences were found in morning serum cortisol and 24-h UFC between the two groups. The CS group exhibits severe hypercortisolemia, demonstrating lower morning plasma ACTH levels and significantly elevated cortisol-related levels compared to both the NFA and MACS groups. (all *P*<0.05) (**Table 1**).

**Table 1:** Demographics, metabolic characteristics, and hormone levels of patients in the NFA, MACS, and CS groups.

	NFA (n = 59)	MACS (n = 36)	CS (n = 20)	*P* value
Demographic factors				
Age (years)	45.9 ± 10.4	48.0 ± 8.5	41.9 ± 12.1	0.103
Sex (male/female)	28/31	11/25	2/18^b^	0.008
Education (years)	13.6 ± 3.3	12.4 ± 3.1	13.4 ± 3.5	0.213
BMI (kg/m^2^)	26.0 ± 4.4	24.8 ± 2.5	26.5 ± 4.8	0.231
Tumor size, cm	1.96 ± 1.00	2.30 ± 0.71	2.64 ± 0.37^b^	0.006
Comorbidities				
Overweight/obesity, n (%)	34 (57.6)	17 (47.2)	16 (80.0)	0.058
IGT/T2DM, n (%)	26 (44.1)	20 (55.6)	13 (65.0)	0.223
Hypertension, n (%)	33 (55.9)	20 (55.6)	13 (65.0)	0.750
Dyslipidemia, n (%)	30 (50.8)	17 (47.2)	14 (70.0)	0.233
Osteopenia/osteoporosis, n (%)	21 (35.6)	18 (50.0)	13 (65.0)	0.058
Hormone levels				
Morning plasma ACTH (pmol/L)	4.08 (3.00, 5.46)	1.78 (1.11, 4.64)^a^	1.11 (1.11, 1.65)^bc^	<0.001
Morning serum cortisol (nmol/L)	404.4 ± 151.2	458.0 ± 170.5	567.6 ± 159.5^bc^	0.001
Midnight serum cortisol (nmol/L)	57.4 (51.3, 94.0)	153.8 (108.5, 225.3)^a^	554.6 (419.0, 620.6)^bc^	<0.001
24-h UFC (nmol/24 h)	691.7 (398.1, 901.1)	694.7 (413.5, 972.6)	1054.5 (588.3, 1295.3)^bc^	0.016
Cortisol after 1 mg DST (nmol/L)	27.6 (24.5, 36.1)	130.7 (94.2, 221.9)	N/A	<0.001
Cortisol after LDDST (nmol/L)	N/A	N/A	601.1 (403.5, 689.5)	N/A
DHEAS (μg/dL)	129.7 (81.9, 187.3)	48.6 (34.4, 86.1)^a^	17.2 (9.23, 52.5)^b^	<0.001

Values are presented as the mean ± standard deviation or number (%).

NFA, nonfunctional adrenal adenoma; MACS, mild autonomous cortisol secretion; CS, Cushing’s syndrome; BMI, body mass index; IGR, impaired glucose regulation; T2DM, type 2 diabetes mellitus; ACTH, adrenocorticotropic hormone; UFC, urinary free cortisol; DST, dexamethasone suppression test; LDDST, low-dose dexamethasone suppression test; DHEAS, dehydroepiandrosterone sulfate; N/A, not available.

^a^P<0.05 for MACS vs. NFA.

^b^P<0.05 for CS vs. NFA.

^c^P<0.05 for MACS vs. CS.

### 3.2 Cognitive function in the NFA, MACS, and CS groups

**Table 2** summarizes the cognitive function of the three groups. The proportion of subjective memory complaints in the MACS and CS groups was higher than in the NFA group, and no statistical difference was found between the MACS and CS groups. In terms of global cognition, the MMSE scores of the three groups were comparable. However, the CS group showed lower MoCA scores than the MACS and NFA groups. The total RNABS scores of the MACS and CS groups were lower than that of the NFA group (all *P*<0.05), but there was no significant difference between the CS and MACS groups. It should be noted that both the MACS and CS groups exhibited a greater proportion of MCI than the NFA group (MACS: 19.4% vs. NFA: 3.4%, CS: 35.0% vs. NFA: 3.4%; all *P*<0.05). The CS group had a slightly higher proportion of MCI than the MACS group, but this difference was not statistically significant (**Table 2**).

**Table 2:** Cognitive function of patients in the NFA, MACS, and CS groups.

	NFA (n = 59)	MACS (n = 36)	CS (n = 20)	P*	MACS vs. NFA*	CS vs. NFA*	MACS vs. CS*
Subjective memory complaints, n (%)	12 (20.3)	15 (41.7)	12 (60.0)	0.003	0.025	0.001	0.188
Global cognition							
MMSE	29.5 ± 0.7	29.3 ± 1.0	29.5 ± 0.8	0.748	1.000	1.000	1.000
MoCA	28.0 ± 1.3	27.4 ± 1.6	26.5 ± 2.0	<0.001	0.545	<0.001	0.014
Total RBANS scores	106.1 ± 10.6	98.3 ± 10.8	95.8 ± 13.0	<0.001	0.006	<0.001	0.176
MCI, n (%)	2 (3.4)	7 (19.4)	7 (35.0)	0.001	0.024	<0.001	0.198
Cognitive subdomains							
Immediate memory	91.9 ± 13.0	82.8 ± 14.5	79.6 ± 15.2	<0.001	0.025	<0.001	0.239
Visuospatial/constructional	109.4 ± 11.3	101.8 ± 8.9	100.4 ± 14.1	0.006	0.017	0.041	1.000
Language	105.0 ± 10.1	103.2 ± 10.0	103.1 ± 15.3	0.091	1.000	0.087	0.357
Attention	117.4 ± 11.8	113.5 ± 10.8	110.7 ± 15.4	0.010	0.637	0.008	0.149
Delayed memory	99.0 ± 7.6	94.2 ± 11.2	91.5 ± 14.4	0.028	0.208	0.043	0.992
Working memory	15.5 ± 1.9	14.3 ± 1.7	14.3 ± 2.1	0.004	0.029	0.014	1.000
Executive function (s)	50.5 ± 14.5	57.4 ± 17.7	54.1 ± 14.0	0.110	0.113	1.000	1.000
Processing speed (s)	71.9 ± 26.7	89.3 ± 42.7	92.3 ± 41.7	<0.001	0.170	0.001	0.130

Values are presented as the mean ± standard deviation or number (%).

NFA, nonfunctional adrenal adenoma; MACS, mild autonomous cortisol secretion; CS, Cushing’s syndrome; MMSE, Mini-Mental State Examination; MoCA, Montreal Cognitive Assessment; RBANS, Repeatable Battery for the Assessment of Neuropsychological Status; MCI, mild cognitive impairment.

^*^P values derived from covariance (ANCOVA) adjusted for age, sex, and years of education and after applying the post-hoc Bonferroni correction.

**Figure 2:**
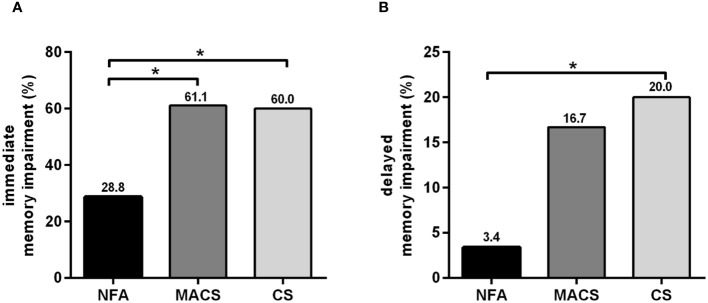
Percentages of memory impairment among patients with NFA, MACS, and CS. **(A)** Percentages of immediate memory impairment in patients with NFA, MACS, and CS; **(B)** Percentages of delayed memory impairment in patients with NFA, MACS, and CS. NFA, nonfunctional adrenal adenoma; MACS, mild autonomous cortisol secretion; CS, Cushing’s syndrome. ^*^ *P*<0.01: Significance compared within the three groups.

Evaluation of cognitive subdomains showed that immediate memory, visuospatial/constructional, and working memory scores were significantly lower in the MACS and CS groups than in the NFA group (all *P*<0.05), but the multiple cognitive domain scores between the MACS and CS groups were comparable. The MACS and CS groups showed a higher proportion of immediate memory impairment than the NFA group (MACS: 61.1% vs. NFA: 28.8%, CS: 60.0% vs. NFA: 28.8%; all *P*<0.05), and no statistically significant difference was found between the MACS and CS groups (**Figure 2A**). Furthermore, the CS group showed poorer performance on attention, delayed memory, and processing speed than the NFA group (all *P*<0.05). There was no statistical difference in language, and executive function scores among the three groups. However, the proportion of delayed memory impairment in the CS group was higher than that in the NFA group (20.0% vs. 3.4%, *P*<0.05), which was comparable to that in the MACS group (**Figure 2B**).

### 3.3 Increased risk of cognitive impairment in patients with MACS and CS

Logistic regression models were fitted to explore immediate and delayed memory impairment risk in patients with MACS and CS versus patients with NFA. Taking NFA as the reference group, the MACS and CS groups had a significantly higher risk of immediate memory impairment in Model 1 (MACS: OR=3.780 [1.365, 10.467], *P*=0.010; CS: OR=6.092 [1.579, 23.507], *P*=0.009) after adjusting for age, sex, and years of education. After further adjusting for overweight/obesity, IGR/T2DM, hypertension, and hyperlipemia in Model 2, the significance of this association persisted (MACS: OR=3.738 [1.329, 10.515], *P*=0.012; CS: OR=6.026 [1.411, 25.730], *P*=0.015). Moreover, the risk of delayed memory impairment was markedly higher in the CS group than the NFA group (Model 1: OR=8.473 [1.029, 69.765], *P*=0.047). The statistical significance still remained in Model 2. Nevertheless, the risk of delayed memory impairment was significantly associated with MACS after adjustment for age, sex, and years of education, and no statistically significant disparity was found after additional adjustment for confounding factors (**Table 3**).

**Table 3:** The risk of immediate and delayed memory impairment in patients with MACS and CS.

	Model 1		Model 2	
	OR (95% CI)	*P*	OR (95% CI)	*P*
Immediate memory impairment				
NFA	1.00		1.00	
MACS	3.780 (1.365, 10.467)	0.010	3.738 (1.329, 10.515)	0.012
CS	6.092 (1.579, 23.507)	0.009	6.026 (1.411, 25.730)	0.015
Delayed memory impairment				
NFA	1.00		1.00	
MACS	7.120 (1.021, 49.644)	0.048	4.401 (0.607, 31.919)	0.143
CS	8.473 (1.029, 69.765)	0.047	12.654 (1.132, 141.455)	0.039

Model 1: adjusted for age, sex, and years of education.

Model 2: adjusted for age, sex, years of education, overweight/obesity (yes/no), impaired glucose regulation/type 2 diabetes mellitus (yes/no), hypertension (yes/no), and hyperlipemia (yes/no).

Odds ratios (ORs) and 95% confidence intervals (CIs) are reported.

NFA, nonfunctional adrenal adenoma; MACS, mild autonomous cortisol secretion; CS, Cushing’s syndrome.

### 3.4 Changes of cognitive function in patients with MACS after surgical/conservative treatment

**Figure 3:**
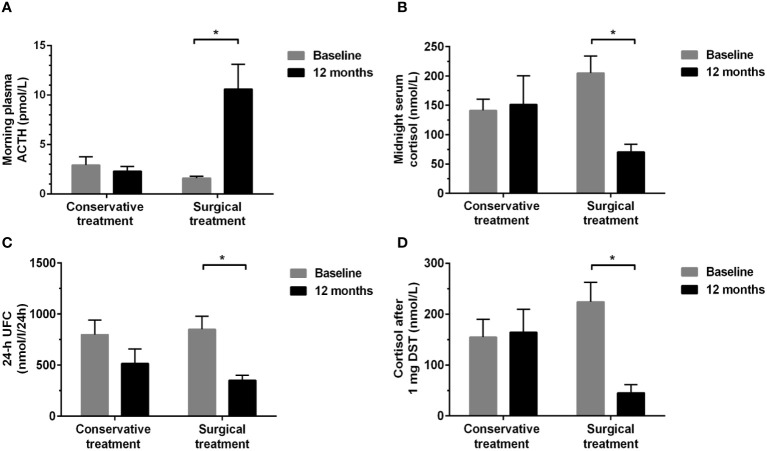
Changes in hormonal levels between baseline and 12-month follow-up in the conservative and surgical treatment groups. **(A)** morning plasma ACTH; **(B)** midnight serum cortisol; **(C)** 24-h UFC; **(D)** serum cortisol levels after 1 mg DST. ACTH, adrenocorticotropic hormone; UFC, urinary free cortisol; DST, dexamethasone suppression test. ^*^P<0.01: Significance compared within groups between baseline and 12-month follow-up.

Seventeen patients with MACS, of whom eleven underwent unilateral adrenalectomy and six received conservative treatment, were re-evaluated for cognitive function after a 12-month follow-up period. There was no significant difference in baseline data among the surgical and conservative treatment groups (**Table 4**; **Supplementary Table 1**). The hypothalamic-pituitary-adrenal axis of all MACS patients with surgical treatment returned to normal within 6 months after surgery, and the mean duration of glucocorticoid replacement therapy in these patients was 2.00 (0.50, 4.13) months. As suspected, surgical treatment could correct cortisol hormone abnormalities, whereas conservative treatment did not show any significant changes in cortisol-related hormone levels during the 12-month follow-up period. Compared to baseline, a significant increase in plasma ACTH levels and a reduction in midnight serum cortisol, 24-h UFC levels, and cortisol levels after 1 mg DST were detected in surgical treatment group after operation (all *P*<0.05). No significant changes were found in the conservative treatment group (**Figures 3A–D**).

**Table 4:** Comparison of cognitive function between baseline and 12-month follow-up after surgical/conservative treatment in patients with MACS.

	Conservative treatment (n=6)			Surgical treatment (n=11)			P^*^	*P^#^*
	Pre-follow-up	Post-follow-up	*P* value	Pre-surgery	Post-surgery	*P* value		
Demographic factors								
Age (years)	53.2 ± 11.7			47.4 ± 8.6			0.289	
Sex (male/female)	2/4			5/6			1.000	
Education (years)	14.5 ± 3.1		N/A	11.9 ± 2.6			0.087	
Global cognition								
MMSE	29.0 ± 1.1	28.5 ± 1.4	0.203	29.6 ± 0.5	29.5 ± 0.9	0.506	0.117	0.726
MoCA	27.2 ± 1.3	26.2 ± 1.5	0.012	27.5 ± 1.8	27.8 ± 1.8	0.397	0.732	0.062
Total RBANS score	98.5 ± 11.1	97.3 ± 10.6	0.302	97.2 ± 14.3	107.0 ± 13.8	0.003	0.848	0.035
MCI (no/yes)	5/1	4/2		8/3	9/2	1.000	1.000	N/A
Cognitive subdomains								
Immediate memory	81.8 ± 14.4	84.3 ± 15.2	0.396	83.3 ± 18.9	94.1 ± 18.1	0.012	0.874	0.282
Visuospatial/constructional	93.7 ± 12.3	96.2 ± 12.3	0.232	102.2 ± 9.8	113.0 ± 9.2	0.006	0.139	0.033
Language	104.2 ± 10.2	100.5 ± 7.9	0.573	100.6 ± 10.6	103.4 ± 11.0	0.085	0.506	0.666
Attention	118.8 ± 7.9	117.7 ± 5.5	0.677	111.1 ± 11.2	111.3 ± 9.7	0.899	0.155	0.211
Delayed memory	97.5 ± 4.5	93.2 ± 11.0	0.245	93.1 ± 14.5	104.1 ± 10.4	0.007	0.484	0.025
Working memory	14.2 ± 1.7	14.0 ± 2.0	0.867	13.7 ± 1.3	14.8 ± 2.1	0.052	0.568	0.751
Executive function (s)	58.0 ± 14.4	59.6 ± 14.7	0.694	65.7 ± 32.4	58.4 ± 23.9	0.174	0.592	0.579
Processing speed (s)	72.4 ± 26.5	75.2 ± 33.2	0.577	106.8 ± 47.6	104.8 ± 62.6	0.795	0.126	0.596
Immediate memory impairment (no/yes)	2/4	3/3	1.000	5/6	8/3	0.620	1.000	N/A
Delayed memory impairment (no/yes)	6/0	3/3	0.182	9/2	10/1	1.000	0.515	N/A

Data are presented as the mean ± standard deviation, or as number.

MACS, mild autonomous cortisol secretion; MMSE, Mini-Mental State Examination; MoCA, Montreal Cognitive Assessment; RBANS, Repeatable Battery for the Assessment of Neuropsychological Status; MCI, mild cognitive impairment; N/A, not available.

^*^P, difference between conservative treatment group and surgical treatment group at baseline.

^#^P, difference between conservative treatment group and surgical treatment group after 12-month follow up, and analyzed by ANCOVA and post hoctest with baseline values and age adjusted.

Except for a decline in MoCA scores from baseline (26.2 ± 1.5 vs. 27.2 ± 1.3, *P*=0.012), the conservative treatment group showed no significant alterations in cognitive function after a 12-month follow-up period. It is worth noting that three patients developed delayed memory impairment in conservative treatment group after 12-month follow-up (**Table 4**). Of note, the total RBANS scores (107.0 ± 13.8 vs. 97.2 ± 14.3), immediate memory (94.1 ± 18.1 vs. 83.3 ± 18.9), visuospatial/constructional (113.0 ± 9.2 vs. 102.2 ± 9.8), and delayed memory scores (104.1 ± 10.4 vs. 93.1 ± 14.5) were significantly improved at 12 months follow-up compared with pre-operation in the surgical treatment group (all *P*<0.05) (**Table 4**). Furthermore, after adjusting for baseline values and age, the improvement of total RBANS scores, visuospatial/constructional and delayed memory scores in the surgical treatment group were greater than that in the conservative treatment group at 12 months follow-up (all *P*<0.05) (**Table 4**). Three patients with immediate and one patient with delayed memory impairment returned to normalcy after surgery. The MMSE, MoCA, and other cognitive subdomains were not markedly changed by surgical treatment between baseline and 12 months follow-up (**Table 4**).

## 4 Discussion

To the best of our knowledge, this is the first prospective study to provide a detailed assessment of cognitive function in patients with MACS and CS. The results extended our initial finding (11), and showed that patients with MACS performed worse than patients with NFA in multiple cognitive domains. Furthermore, compared to patients with CS, those with MACS showed mild hormonal and metabolic disturbances, but the cognitive impairment of both groups was comparable. Interestingly, we first reported that some cognitive domains improved in a subset of MACS patients one year after remission of hypercortisolism.

Glucocorticoids are believed to have a substantial effect on brain function, with implications for mood, stress, and cognitive functioning (23). Exogenous or endogenous hypercortisolism have been shown to lead to brain deterioration and cognitive impairment in previous studies (24, 25). CS is a severe, chronic, and life-threatening disease caused by prolonged endogenous hypercortisolism (26). The relationship between cognitive impairment and CS have been well documented (27), with memory being the most frequently affected domain (7). Our findings are consistent with the above, in that we found that 60% patients with CS had memory-related complaints, and about three-fifths of CS patients exhibited immediate memory impairment, and their cognitive performance was notably deficient in various cognitive domains. The present study provides rigorous evidence about the relationship between excessive cortisol secretion and cognitive impairment.

Recently, several studies have focused on cognitive function in patients with MACS, but the findings have been inconsistent (11, 28, 29). Early research suggested that patients with MACS performed better in some cognitive domains (29). However, a notable limitation of the study was its small sample size, encompassing only 16 patients with MACS. Among them, only three patients displayed a serum cortisol level ≥138 nmol/L after a 1 mg DST (29). In a recently published prospective study, we first reported cognitive impairment in MACS patients. The present study with a relatively larger sample size has shown consistent results with our primary observation (11). In addition, our study revealed that patients with MACS were associated with an increased risk of immediate memory impairment. Sulu et al. recently observed a decreased trend in language domain of cognition among patients with MACS, while other cognitive domains remained unaffected (28). Furthermore, they also detected a reduction in the left thalamus volumes in patients with MACS for the first time (28). Overall, the present study confirmed our previous findings wherein mild endogenous cortisol secretion had a negative effect on cognitive function.

To date, very few studies have carried out direct comparisons of cognitive function between patients with MACS and CS (28). Previous studies have reported increased cortisol levels being linked to faster cognitive deterioration in aged human or patients with Alzheimer’s disease (30–32) and a dose-dependent relationship between supra-physiological doses of glucocorticoids and cognitive function (33). Therefore, it is speculated that cognitive impairment may be less severe in MACS patients than in CS patients. Different from previous assumptions, this study found comparable impaired global cognitive function and multiple cognitive subdomains between patients with MACS and CS. Additionally, we found that both groups showed a similar proportion of immediate and delayed memory impairment. Given that cognitive impairment is directly related to both cortisol levels and duration of hypercortisolism (33). Compared to CS, patients with MACS might have longer duration of hypercortisolism although with mildly elevated cortisol levels, owing to the absence of symptoms and signs of overt hypercortisolism. This might be the reason for comparable cognitive impairment in patients with MACS and CS. The present findings reinforce that attention should be paid to the risk of cognitive impairment in patients with MACS, and the cognitive evaluation of MACS should be emphasized in clinical practice.

Surgery is the primary approach to correcting hypercortisolism in MACS. Multiple studies have demonstrated the beneficial effect of adrenalectomy on cardiovascular risk factors in patients with MACS (34). However, the change in cognitive function of MACS patients after adrenalectomy has not been evaluated. In our study, we found that the total RBANS scores, visuospatial/constructional, immediate and delayed memory scores were significantly improved at 12 months follow-up compared with pre-operation in the surgical treatment group. Nevertheless, in the conservative treatment group, no significant changes were found in multiple cognitive domains after a 12-month follow-up, with some cognitive function scores even showing further deterioration. Therefore, we believe that our results are among the first to confirm that surgical treatment can partially ameliorate cognitive impairment in patients with MACS. Until now, there is no consensus regarding the optimal management strategy (surgery or conservative management) for MACS. Given the lack of high-quality randomized trials confirming the clinical benefit of surgery, current guidelines recommend an individualized approach instead of surgery for all patients with MACS (1). Assessment of cortisol-related metabolic comorbidities is an important aspect when considering a surgical approach for MACS (1). This study provides new evidence and recommends the inclusion of cognitive function assessment in the preoperative evaluation of MACS. In addition, our research also found that impaired cognitive function in patients with MACS was not completely restored to normal. This finding is consistent with the results obtained in CS patients (9), which suggests that hypercortisolism induces persistent and partially irreversible deficits in mental function. Hence, rapid normalization of hypercortisolism in MACS patients may contribute to alleviation of cognitive dysfunction. Studies with a longer follow-up and larger sample size are needed to verify the postoperative cognitive function improvement in patients with MACS.

This study has some limitations. First, although without significance, the included CS patients have a relatively younger age and a higher female proportion than the MACS group, which may affect the results of cognitive function. Whereas, this is the demographic characteristic of patients with CS, the RBANS scale utilized age-adjusted normative scores, and statistical analysis further corrected for age and sex. The above methods mitigate the bias caused by age and sex differences between CS and MACS patients. Second, only half of the MACS patients were re-evaluated for cognitive function, which may have introduced a bias in the results. Third, our study only assessed the changes in cognitive function of patients with MACS based on a 12-month follow-up after surgery, and the impact of long-term remission of hypercortisolism on cognitive function still remains unclear. Further investigations into the long-term outcome of MACS with different treatment are warranted.

## 5 Conclusion

In conclusion, the present study confirmed cognitive impairment in patients with MACS and demonstrated comparable cognitive impairment in patients with MACS and CS. Our study provides novel evidence that remission of hypercortisolemia after surgery can improve cognitive function in patients with MACS. Therefore, in addition to the metabolic complications, cognitive function should be evaluated in MACS patients in clinical practice. More consolidated evidence in a larger population with longer follow-up is further needed to confirm the benefit of surgery to improve cognitive function in MACS.

## Data availability statement

The original contributions presented in the study are included in the article material. Further inquiries can be directed to the corresponding author.

## Ethics statement

The studies involving humans were approved by Ethics Committee of Nanjing Drum Tower Hospital, Affiliated Hospital of Medical School, Nanjing University. The studies were conducted in accordance with the local legislation and institutional requirements. The participants provided their written informed consent to participate in this study. Written informed consent was obtained from the individual(s) for the publication of any potentially identifiable images or data included in this article.

## Author contributions

ML: Data curation, Formal analysis, Methodology, Writing – original draft. WJZ: Methodology, Writing – review & editing. WZ: Data curation, Formal analysis, Writing – review & editing. ZT: Data curation, Writing – review & editing. ZZ: Methodology, Validation, Writing – review & editing. YL: Data curation, Writing – review & editing. ZWZ: Methodology, Writing – review & editing. FY: Supervision, Writing – review & editing. DZ: Funding acquisition, Supervision, Writing – review & editing. PL: Conceptualization, Funding acquisition, Methodology, Project administration, Supervision, Writing – review & editing.

## References

[1] Fassnacht M Arlt W Bancos I Dralle H Newell-Price J Sahdev A. Management of adrenal incidentalomas: European Society of Endocrinology Clinical Practice Guideline in collaboration with the European Network for the Study of Adrenal Tumors. Eur J Endocrinol. (2016) 175:G1–1G34. doi: 10.1530/EJE-16-0467 27390021

[2] Ebbehoj A Li D Kaur RJ Zhang C Singh S Li T. Epidemiology of adrenal tumours in Olmsted County, Minnesota, USA: a population-based cohort study. Lancet Diabetes Endocrinol. (2020) 8:894–902. doi: 10.1016/S2213-8587(20)30314-4 33065059 PMC7601441

[3] Elhassan YS Alahdab F Prete A Delivanis DA Khanna A Prokop L. Natural history of adrenal incidentalomas with and without mild autonomous cortisol excess: A systematic review and meta-analysis. Ann Intern Med. (2019) 171:107–16. doi: 10.7326/M18-3630 31234202

[4] Di Dalmazi G Vicennati V Garelli S Casadio E Rinaldi E Giampalma E. Cardiovascular events and mortality in patients with adrenal incidentalomas that are either non-secreting or associated with intermediate phenotype or subclinical Cushing's syndrome: a 15-year retrospective study. Lancet Diabetes Endocrinol. (2014) 2:396–405. doi: 10.1016/S2213-8587(13)70211-0 24795253

[5] Deutschbein T Reimondo G Di Dalmazi G Bancos I Patrova J Vassiliadi DA. Age-dependent and sex-dependent disparity in mortality in patients with adrenal incidentalomas and autonomous cortisol secretion: an international, retrospective, cohort study. Lancet Diabetes Endocrinol. (2022) 10(7):499–508. doi: 10.1016/S2213-8587(22)00100-0 PMC9679334 35533704

[6] Frimodt-Møller KE Møllegaard Jepsen JR Feldt-Rasmussen U Krogh J. Hippocampal volume, cognitive functions, depression, anxiety, and quality of life in patients with cushing syndrome. J Clin Endocrinol Metab. (2019) 104:4563–77. doi: 10.1210/jc.2019-00749 31215997

[7] Piasecka M Papakokkinou E Valassi E Santos A Webb SM de Vries F. Psychiatric and neurocognitive consequences of endogenous hypercortisolism. J Intern Med. (2020) 288:168–82. doi: 10.1111/joim.13056 32181937

[8] Na S Fernandes MA Ioachimescu AG Penna S. Neuropsychological and emotional functioning in patients with cushing's syndrome. Behav Neurol. (2020) 2020:4064370. doi: 10.1155/2020/4064370 32831970 PMC7428886

[9] Broersen L Andela CD Dekkers OM Pereira AM Biermasz NR. Improvement but no normalization of quality of life and cognitive functioning after treatment of cushing syndrome. J Clin Endocrinol Metab. (2019) 104:5325–37. doi: 10.1210/jc.2019-01054 31276166

[10] Forget H Lacroix A Bourdeau I Cohen H. Long-term cognitive effects of glucocorticoid excess in Cushing's syndrome. Psychoneuroendocrinology. (2016) 65:26–33. doi: 10.1016/j.psyneuen.2015.11.020 26708069

[11] Liu MS Tian ZY Zhang Z Yang F Lou Y Wang YJ. Impaired cognitive function in patients with autonomous cortisol secretion in adrenal incidentalomas. J Clin Endocrinol Metab. (2023) 108:633–41. doi: 10.1210/clinem/dgac603 36263685

[12] Nieman LK Biller BM Findling JW Newell-Price J Savage MO Stewart PM. The diagnosis of Cushing's syndrome: an Endocrine Society Clinical Practice Guideline. J Clin Endocrinol Metab. (2008) 93:1526–40. doi: 10.1210/jc.2008-0125 PMC2386281 18334580

[13] WHO Expert Consultation. Appropriate body-mass index for Asian populations and its implications for policy and intervention strategies. Lancet. (2004) 363:157–63. doi: 10.1016/S0140-6736(03)15268-3 14726171

[14] Alberti KG Zimmet PZ. Definition, diagnosis and classification of diabetes mellitus and its complications. Part 1: diagnosis and classification of diabetes mellitus provisional report of a WHO consultation. Diabetes Med. (1998) 15:539–53. doi: 10.1002/(SICI)1096-9136(199807)15:7<539::AID-DIA668>3.0.CO;2-S 9686693

[15] Perk J De Backer G Gohlke H Graham I Reiner Z Verschuren WM. European Guidelines on cardiovascular disease prevention in clinical practice (version 2012): The Fifth Joint Task Force of the European Society of Cardiology and Other Societies on Cardiovascular Disease Prevention in Clinical Practice (constituted by representatives of nine societies and by invited experts). Atherosclerosis. (2012) 223:1–68. doi: 10.1016/j.atherosclerosis.2012.05.007 22698795

[16] Sereg M Szappanos A Toke J Karlinger K Feldman K Kaszper E. Atherosclerotic risk factors and complications in patients with non-functioning adrenal adenomas treated with or without adrenalectomy: a long-term follow-up study. Eur J Endocrinol. (2009) 160:647–55. doi: 10.1530/EJE-08-0707 19174533

[17] Camacho PM Petak SM Binkley N Diab DL Eldeiry LS Farooki A. American association of clinical endocrinologists/american college of endocrinology clinical practice guidelines for the diagnosis and treatment of postmenopausal osteoporosis-2020 update. Endocr Pract. (2020) 26:1–46. doi: 10.4158/GL-2020-0524SUPPL 32427503

[18] Jia X Wang Z Huang F Su C Du W Jiang H. A comparison of the Mini-Mental State Examination (MMSE) with the Montreal Cognitive Assessment (MoCA) for mild cognitive impairment screening in Chinese middle-aged and older population: a cross-sectional study. BMC Psychiatry. (2021) 21:485. doi: 10.1186/s12888-021-03495-6 34607584 PMC8489046

[19] Randolph C Tierney MC Mohr E Chase TN. The Repeatable Battery for the Assessment of Neuropsychological Status (RBANS): preliminary clinical validity. J Clin Exp Neuropsychol. (1998) 20:310–9. doi: 10.1076/jcen.20.3.310.823 9845158

[20] Karantzoulis S Novitski J Gold M Randolph C. The Repeatable Battery for the Assessment of Neuropsychological Status (RBANS): Utility in detection and characterization of mild cognitive impairment due to Alzheimer's disease. Arch Clin Neuropsychol. (2013) 28:837–44. doi: 10.1093/arclin/act057 23867976

[21] Nasreddine ZS Phillips NA Bédirian V Charbonneau S Whitehead V Collin I. The Montreal Cognitive Assessment, MoCA: a brief screening tool for mild cognitive impairment. J Am Geriatr Soc. (2005) 53:695–9. doi: 10.1111/j.1532-5415.2005.53221.x 15817019

[22] Egan MF Kost J Voss T Mukai Y Aisen PS Cummings JL. Randomized trial of verubecestat for prodromal alzheimer's disease. N Engl J Med. (2019) 380:1408–20. doi: 10.1056/NEJMoa1812840 PMC6776078 30970186

[23] Joëls M. Corticosteroids and the brain. J Endocrinol. (2018) 238:R121–121R130. doi: 10.1530/JOE-18-0226 29875162

[24] Tatomir A Micu C Crivii C. The impact of stress and glucocorticoids on memory. Clujul Med. (2014) 87:3–6. doi: 10.15386/cjm.2014.8872.871.at1cm2 26527987 PMC4462413

[25] Ouanes S Popp J. High cortisol and the risk of dementia and alzheimer's disease: A review of the literature. Front Aging Neurosci. (2019) 11:43. doi: 10.3389/fnagi.2019.00043 30881301 PMC6405479

[26] Barbot M Zilio M Scaroni C. Cushing's syndrome: Overview of clinical presentation, diagnostic tools and complications. Best Pract Res Clin Endocrinol Metab. (2020) 34:101380. doi: 10.1016/j.beem.2020.101380 32165101

[27] Bernini G Tricò D. Cushing's syndrome and steroid dementia. Recent Pat Endocr Metab Immune Drug Discov. (2016) 10:50–5. doi: 10.2174/1872214810666160809113021 27515536

[28] Sulu C Koca O Icli TB Oz A Kargin OA Durcan E. Altered thalamic volume in patients with mild autonomous cortisol secretion: a structural brain MRI study. Neuroradiology. (2023) 65:1037–51. doi: 10.1007/s00234-023-03156-3 37121916

[29] Morelli V Ghielmetti A Caldiroli A Grassi S Siri FM Caletti E. Mental health in patients with adrenal incidentalomas: is there a relation with different degrees of cortisol secretion. J Clin Endocrinol Metab. (2021) 106:e130–130e139. doi: 10.1210/clinem/dgaa695 33017843 PMC7765655

[30] Zheng B Tal R Yang Z Middleton L Udeh-Momoh C. Cortisol hypersecretion and the risk of Alzheimer's disease: A systematic review and meta-analysis. Ageing Res Rev. (2020) 64:101171. doi: 10.1016/j.arr.2020.101171 32971258

[31] Popp J Wolfsgruber S Heuser I Peters O Hüll M Schröder J. Cerebrospinal fluid cortisol and clinical disease progression in MCI and dementia of Alzheimer's type. Neurobiol Aging. (2015) 36:601–7. doi: 10.1016/j.neurobiolaging.2014.10.031 25435336

[32] Lupien SJ de Leon M de Santi S Convit A Tarshish C Nair NP. Cortisol levels during human aging predict hippocampal atrophy and memory deficits. Nat Neurosci. (1998) 1:69–73. doi: 10.1038/271 10195112

[33] De Alcubierre D Ferrari D Mauro G Isidori AM Tomlinson JW Pofi R. Glucocorticoids and cognitive function: a walkthrough in endogenous and exogenous alterations. J Endocrinol Invest. (2023) 46(10):1961–82. doi: 10.1007/s40618-023-02091-7 PMC10514174 37058223

[34] Bancos I Alahdab F Crowley RK Chortis V Delivanis DA Erickson D. THERAPY OF ENDOCRINE DISEASE: Improvement of cardiovascular risk factors after adrenalectomy in patients with adrenal tumors and subclinical Cushing's syndrome: a systematic review and meta-analysis. Eur J Endocrinol. (2016) 175:R283–283R295. doi: 10.1530/EJE-16-0465 27450696

