# Supplementary material for: Comparable cognitive impairment was detected in MACS and CS and alleviated after remission of hypercortisolism in MACS
Source: Front Endocrinol (Lausanne). 2024 Jul 31;15:1373101. doi: 10.3389/fendo.2024.1373101 (PMC11322108; doi:10.3389/fendo.2024.1373101)
Supplement: Supplementary file 1 [file Table_1.docx]

**Comparable cognitive impairment was detected in MACS and CS and alleviated after remission of hypercortisolism in MACS**

**Mengsi Liu ^1,2,3,^****^†^,** **Wenji Zhao ^1,3†^, Wei Zhang^1,3,†^, Zhaoyang Tian ^1,3^, Zhou Zhang ^1,3^,Yuan Lou** **^1,3^, Ziwei Zhang ^1,3^, Fan Yang ^1,3^, Dalong Zhu ^1,2,3^ and Ping Li ^1,3,*^**

^1^Department of Endocrinology, Endocrine and Metabolic Disease Medical Center, Nanjing Drum Tower Hospital, Affiliated Hospital of Medical School, Nanjing University, Nanjing 210008, China

^2^Department of Endocrinology, Endocrine and Metabolic Disease Medical Center, Nanjing Drum Tower Hospital, Chinese Academy of Medical Sciences & Peking Union Medical College, Graduate School of Peking Union Medical College, Nanjing 210008, China

^3^Branch of National Clinical Research Centre for Metabolic Diseases, Nanjing, China

*** Correspondence:**Corresponding Author: Ping Li
[liping@nju.edu.cn](mailto:liping@nju.edu.cn)

**^†^**These authors contributed equally to this work and share first authorship.

Supplementary Table 1. Metabolic characteristics, and hormone levels at baseline in the conservative and surgical treatment groups.

|  | Conservative treatment  (n=6) | Surgical treatment  (n=11) | *P* |
| --- | --- | --- | --- |
| Metabolic index |  |  |  |
| BMI (kg/m^2^) | 24.5 ± 1.1 | 24.7 ± 1.9 | 0.808 |
| Systolic blood pressure (mmHg) | 132.8 ± 18.7 | 128.9 ± 14.0 | 0.630 |
| Diastolic blood pressure (mmHg) | 80.3 ± 12.1 | 85.6 ± 8.8 | 0.315 |
| Fasting glucose (mmol/L) | 5.24 ± 0.86 | 5.03 ± 1.30 | 0.733 |
| HbA_1c_ (%) | 5.57 ± 0.29 | 5.91 ± 1.19 | 0.503 |
| TG (mmol/L) | 1.38 ± 0.51 | 1.98 ± 1.15 | 0.246 |
| TC (mmol/L) | 4.96 ± 1.24 | 4.64 ± 0.43 | 0.570 |
| HDL-C (mmol/L) | 1.29 ± 0.29 | 1.06 ± 0.16 | 0.053 |
| LDL-C (mmol/L) | 2.90 ± 0.86 | 2.75 ± 0.41 | 0.690 |
| Comorbidities |  |  |  |
| Overweight/obesity, n (%) | 3 (50.0) | 6 (54.5) | 1.000 |
| IGT/T2DM, n (%) | 2 (33.3) | 5 (45.5) | 1.000 |
| Hypertension, n (%) | 4 (66.7) | 5 (45.5) | 0.620 |
| Dyslipidemia, n (%) | 5 (83.3) | 7 (63.6) | 0.600 |
| Osteopenia/osteoporosis, n (%) | 3 (50.0) | 7 (63.6) | 0.644 |
| Hormone levels |  |  |  |
| Morning plasma ACTH (pmol/L) | 2.36 (1.11, 4.82) | 1.52 (1.11, 2.29) | 0.301 |
| Morning serum cortisol (nmol/L) | 368.9 (291.4, 801.6) | 400.5 (331.2, 471.1) | 0.961 |
| Midnight serum cortisol (nmol/L) | 131.4 (99.3, 191.0) | 207.3 (96.8, 293.2) | 0.301 |
| 24-h UFC (nmol/24 h) | 916.9 (464.2, 1074.9) | 811.0 (436.4, 1241.7) | 1.000 |
| Cortisol after 1 mg DST (nmol/L) | 147.1 (75.7, 219.9) | 149.5 (106.4, 360.3) | 0.301 |
| DHEAS (μg/dL) | 57.5 (32.1, 96.4) | 20.2 (15.8, 87.1) | 0.462 |

Data are presented as the mean ± standard deviation, median (25th percentile, 75th percentile) or as number (%).

Abbreviations: BMI, body mass index; HbA1c glycosylated hemoglobin; TC, total cholesterol; TG, triglyceride; HDL-C, high density lipoprotein-cholesterol; LDL-C, low density lipoprotein-cholesterol; IGR, impaired glucose regulation; T2DM, diabetes mellitus type 2; ACTH, adrenocorticotropic hormone; UFC, urinary free cortisol; DST, dexamethasone suppression test; DHEAS, dehydroepiandrosterone sulfate.
